# Dataset for the interfacial tension and phase properties of the ternary system water – 2-butoxyethanol – toluene

**DOI:** 10.1016/j.dib.2021.107532

**Published:** 2021-10-30

**Authors:** Alexandra A. Kuchierskaya, Anton P. Semenov, Adeliya R. Sayfutdinova, Dmitry S. Kopitsyn, Vladimir A. Vinokurov, Mikhail A. Anisimov, Andrei A. Novikov

**Affiliations:** a Department of Physical and Colloid Chemistry, Gubkin University, 65/1, Leninsky prospect, 119991 Moscow, Russian Federation; b Department of Chemical and Biomolecular Engineering, University of Maryland, College Park, MD 20742, USA

**Keywords:** Phase diagram, Binodal curve, Solubility, Density, Viscosity, Critical point

## Abstract

Two-phase samples containing water, 2-butoxyethanol, and toluene in the different mass ratios were gravimetrically prepared in the jacketed cells at *T*=293.15 K and *p*=0.100 MPa and equilibrated for 24 h. The samples were volumetrically titrated until homogeneous. Then new samples were prepared in the two-phase region with compositions in the immediate proximity to the expected separation boundary and titrated until homogeneous. The critical point was located, keeping the phase ratio of 1:1 during the titration. The density of homogeneous samples obtained during titration was measured using the density meter. These data were used to construct an interpolation of the density along the separation boundary. New two-phase samples were prepared; the interfacial tension, density, and viscosity were measured. Thus, interfacial tension isotherm and viscosity isotherm were obtained using density interpolation to determine the composition of the equilibrated phases.

The obtained data can be used to prepare the two-phase samples with desired properties, design the oil-water separation processes, and develop new oil spill dispersants containing 2-butoxyethanol.

This article is a co-submission with a paper [1].

## Specifications Table

**Table:** 

Subject	Chemistry
Specific subject area	Surface and colloid chemistry
Type of data	Tables, figures
How the data were acquired	The location of the phase separation boundary on the water – 2-butoxyethanol – toluene diagram was determined by volumetric titration. At first, two-phase samples containing water, 2-butoxyethanol (BEG), and toluene (TOL) in the different mass ratios were gravimetrically prepared using the balances PA413C (Ohaus, USA). The comparison with other ternary systems containing BEG (or MeEG) was conducted using available literature presented in references. The density of homogeneous samples obtained during titration was measured using the density meter DMA 4500 (Anton Paar, Austria). These data were used to construct an interpolation of the density along the separation boundary, which was applied to determine the compositions of equilibrated phases of heterogeneous samples. A spinning drop tensiometer SDT (Krüss, Hamburg, Germany) and a force tensiometer K20 (Krüss, Hamburg, Germany) were used to measure the interfacial tension in the two-phase region. The density and dynamic viscosity of equilibrated phases were measured with a rolling-ball viscometer Lovis 2000 ME (Anton Paar, Austria) integrated with a density meter DMA 4500.
Data format	Raw and analyzed
Parameters for data collection	The samples of the ternary system were prepared in the laboratory.
Description of data collection	Phase diagram, interfacial tension isotherm, density, and viscosity for the ternary system water – 2-butoxyethanol – toluene.
Data source location	Gubkin University, Department of Physical and Colloid Chemistry. Moscow, Russia
Data accessibility	With the article
Related research article	Alexandra A. Kuchierskaya, Anton P. Semenov, Adeliya R. Sayfutdinova, Dmitry S. Kopitsyn, Vladimir A. Vinokurov, Mikhail A. Anisimov, Andrei A. Novikov. Interfacial tension and phase properties of water – hydrotrope – oil solutions: water – 2-butoxyethanol – toluene. Journal of Molecular Liquids 344 (2021) 117683 [1].

## Value of the data

• In this work, we compared behavior of hydrotropes – tertiary butanol (TBA) and 2-butoxyethanol (BEG) – at the oil/water interface. It shows a high degree of universality, however, more hydrophobic BEG demonstrates higher adsorption at the oil/water interface and shifts the critical point closer to the water vertex. These results could be used to predict the behavior of the systems with different hydrotropes.
• The present data on the phase equilibria can be useful in constructing phase diagrams of ternary systems containing low-molecular-weight amphiphiles.
• It has been shown that measuring density along the separation boundary can be used to determine the composition of equilibrium phases in the two-phase region with a high accuracy.
• Data on interfacial tension, density, and viscosity could be used to rationally design oil spill dispersants.

## 1 Data Description

**Fig. 1:**
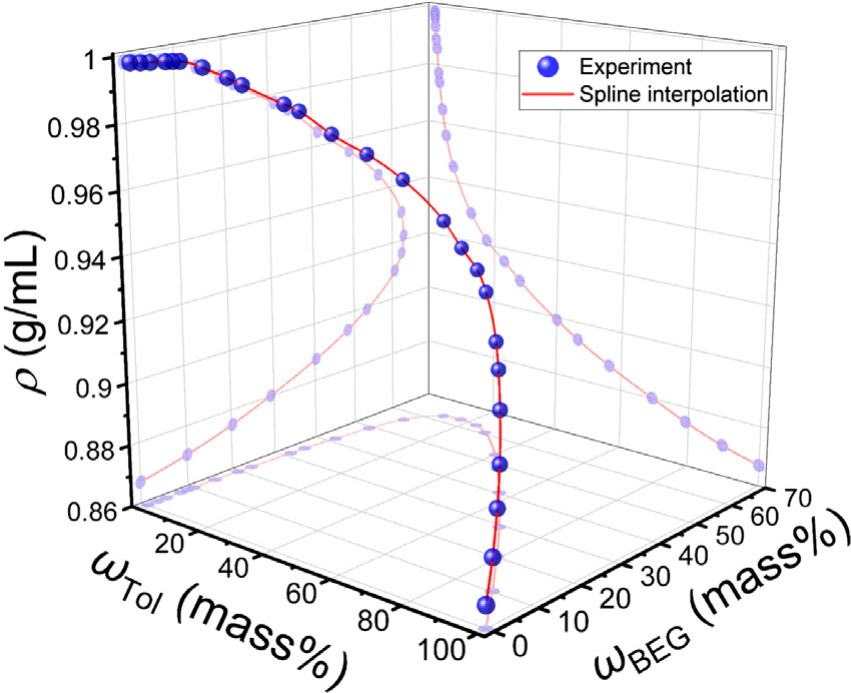
Density at the separation boundary as a function of the composition for the ternary system of H_2_O–BEG–TOL at 293.15 K; spheres – experimental data, red line – cubic spline interpolation, projections of experimental points and interpolation curve on the XY, XZ, YZ planes are shown. Data are from Table 1 (*ω*_Tol_ is from Column VII; *ω*_BEG_ is from Column VI; *ρ* is from Column XIV).

**Fig. 2:**
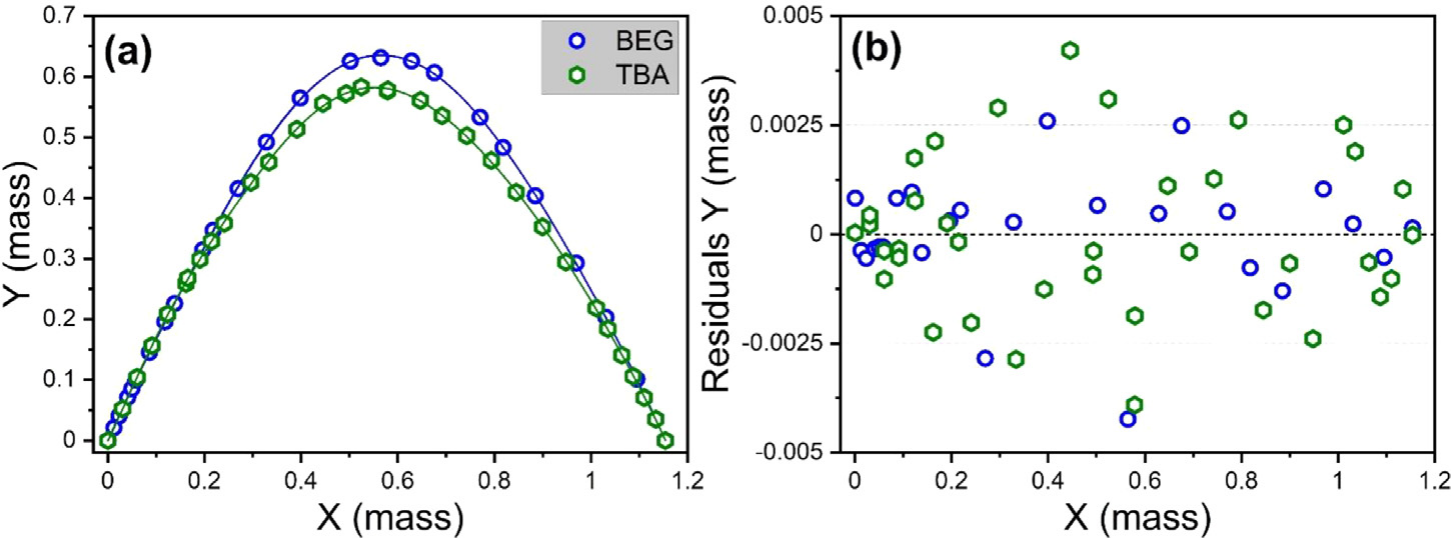
(a) Binodal curves at 293.15 K for ternary systems of H_2_O–TBA–TOL [6] and H_2_O–BEG–TOL (this work) approximated by 9th order polynomial; (b) fitting residuals; (Y is mass fraction of hydrotrope *ω*_h_, $X=\left(2\times \;\omega_{\text{TOL}}\;+\;\omega_{h}\right)/\sqrt{3}$); (a) circles and hexagons are experimental data (titration); blue and green curves are polynomial approximations. Data are from Table 2 (X is from Column II, Y is from Column III, residuals Y are from Column IV).

**Fig. 3:**
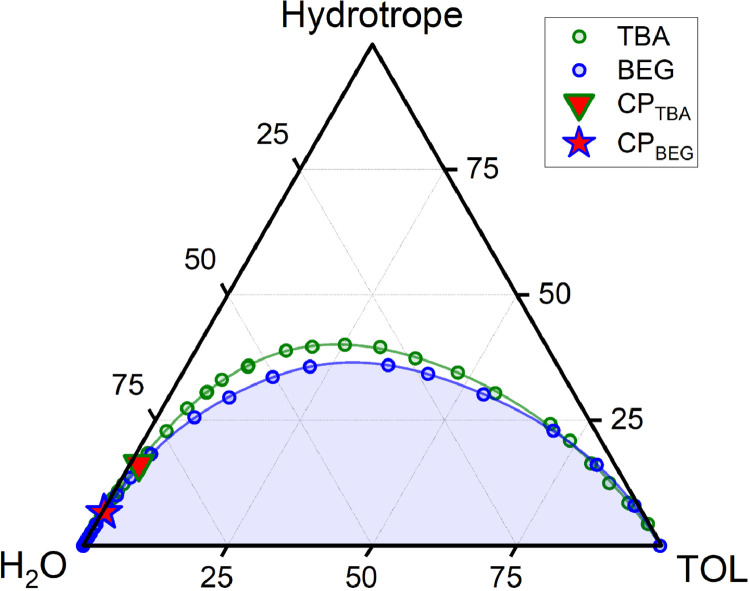
Phase diagrams at 293.15 K of the systems W–BEG–TOL (this work) and W–TBA–TOL [6] in mole percentage; circles are experimental points (titration), color lines are 9th order polynomial approximations; red filled markers denote critical points; blue-filled area corresponds to the two-phase region in the W–BEG–TOL system. Data on W–BEG–TOL system are from Table 1 (*x*_H__2__O_ is from Column XI; *x*_BEG_ is from Column XII; x_Tol_ is from Column XIII).

**Fig. 4:**
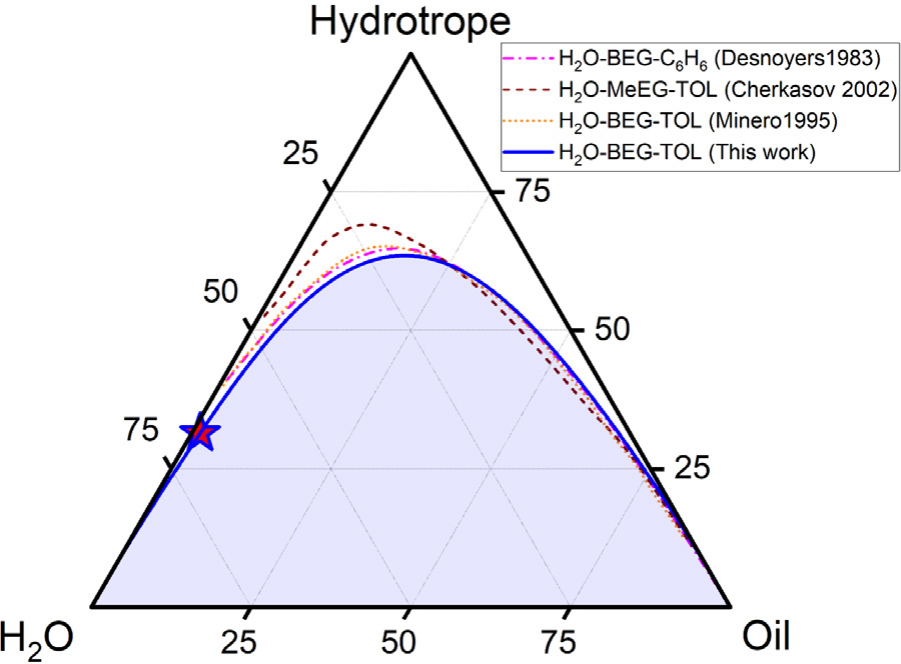
Comparison of data of this work for the system W–BEG–TOL at 293.15 K with literature data for systems W–BEG–benzene at 298.15 K [7], W (pH=3)–BEG–toluene at 298.15 K [8], W–MeEG–toluene at 283.15 K [9] in mass percentage. Data on W–BEG–TOL system are from Table 1 (*ω*_H2O_ is from Column V; *ω*_BEG_ is from Column VI; *ω*_Tol_ is from Column VII).

**Fig. 5:**
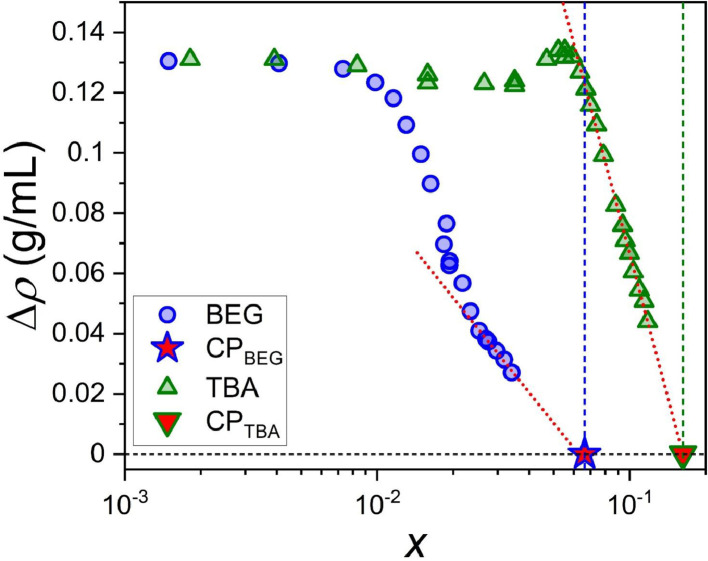
Density difference of the equilibrated phases in the systems W–BEG–TOL (this work) and W–TBA–TOL [6] versus the equilibrium hydrotrope mole fraction in water phase at 293.15 K; red filled symbols are critical points, vertical color dashed lines are critical mole fractions of hydrotropes, red dotted lines are linear approximations of density difference for near-critical samples of W–BEG–TOL and W–TBA–TOL. Data on W–BEG–TOL system are from Tables 3,4 (*X*_BEG_ in water phase is from Table 3, Column VIII; ∆*ρ* is from Table 4, Column XVI).

**Fig. 6:**
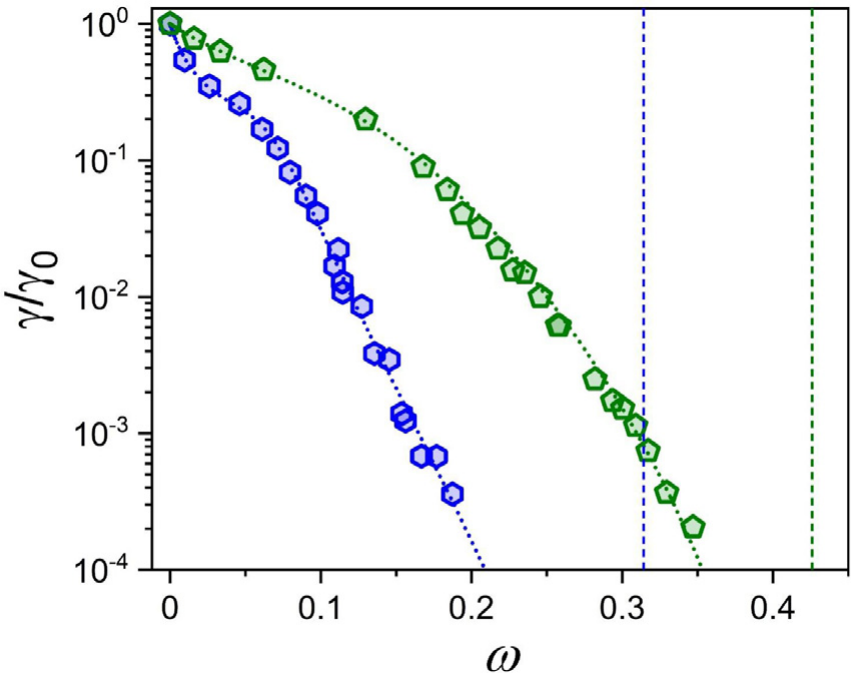
Interfacial tension isotherms at 293.15 K for the W–BEG–TOL (this work) and W–TBA–TOL [6] systems versus the equilibrium hydrotrope mass fraction in water phase; the symbols are experimental data (green pentagons for TBA, blue hexagons for BEG), the dotted curves are crossover approximations [4], the vertical dashed lines indicate the critical hydrotrope concentrations. Data on W–BEG–TOL system are from Table 3 (*ω*_BEG_ in water phase is from Column X; *γ*/*γ*_0_ is from Column XII).

The data on phase equilibrium and interfacial phenomena of the system water – BEG – toluene is reported in this work in accordance with recent IUPAC recommendations [2]. Compositions of two-phase and one-phase samples along the phase separation boundary are presented in Table 1. Data in Table 1 include the densities of water – BEG – toluene homogeneous systems obtained during titration, which were further used to construct an interpolation of the density along the separation boundary (Fig. 1). Approximated binodal curves at 293.15 K for ternary water – BEG – toluene and water – TBA – toluene systems and fitting residuals are shown in Fig. 2 (the data for the Fig. 2 construction are in Table 2). The ternary phase diagrams of water – BEG – toluene and water – TBA – toluene systems at 293.15 K are shown in Fig. 3. The comparison of water – BEG – toluene with other ternary systems containing BEG (or MeEG) is presented in Fig. 4. Compositions of water – BEG – toluene system samples used to construct Figs. 3 and 4 are in Table 1. Table 3 contains the compositions of the two-phase liquid samples, the interfacial tension between the equilibrated phases, and the normalized interfacial tension, which is illustrated by the interfacial tension isotherm shown in Fig. 6. Compositions, density, viscosity of the equilibrated phases, and density difference of the equilibrated phases are reported in Table 4. Fig. 5 shows density difference of the equilibrated phases for ternary systems water – BEG – toluene (Table 4) and water – TBA – toluene.

**Table 1:** . Phase separation boundary and density data on H_2_O–BEG–TOL system at *T*=293.15 K and *p*=0.100 MPa obtained by titration.^a^

	Two-phase points			One-phase points			Two-phase points			One-phase points			
	Mass fraction			Mass fraction			Mole fraction			Mole fraction			
Point number	H_2_O	BEG	TOL	H_2_O	BEG	TOL	H_2_O	BEG	TOL	H_2_O	BEG	TOL	*ρ*, g/mL (one-phase state)
I	II	III	IV	V	VI	VII	VIII	IX	X	XI	XII	XIII	XIV
1	0.99909	0	0.00091	0.99910	0	0.00090	0.99982	0	0.00018	0.99982	0	0.00018	0.99821
2	0.9788	0.0206	0.0006	0.9787	0.0207	0.0006	0.99668	0.00320	0.00012	0.99666	0.00321	0.00013	0.99769
3	0.9595	0.0401	0.0004	0.9595	0.0402	0.0004	0.99359	0.00633	0.00008	0.99359	0.00634	0.00007	0.99729
4	0.9283	0.0713	0.0004	0.9285	0.0711	0.0004	0.98834	0.01157	0.00008	0.98839	0.01154	0.00007	0.99671
5	0.9139	0.0854	0.0006	0.9139	0.0855	0.0006	0.98583	0.01404	0.00013	0.98581	0.01406	0.00013	0.99644
6	0.89954	0.09943	0.00103	0.89958	0.09940	0.00102	0.98321	0.01657	0.00022	0.98322	0.01656	0.00022	0.99613
7	0.85249	0.14519	0.00232	0.85251	0.14518	0.00231	0.97419	0.02529	0.00052	0.97419	0.02529	0.00052	0.99311
8	0.7995	0.1955	0.0050	0.7996	0.1955	0.0049	0.96293	0.03589	0.00118	0.96295	0.03589	0.00116	0.98863
9	0.7673	0.2261	0.0066	0.7671	0.2254	0.0075	0.95547	0.04292	0.00161	0.95538	0.04279	0.00183	0.98567
10	0.6723	0.3144	0.0134	0.6726	0.3141	0.0133	0.93007	0.06630	0.00362	0.93018	0.06623	0.00359	0.97725
11	0.6362	0.3482	0.0156	0.6376	0.3465	0.0159	0.91893	0.07667	0.00441	0.91934	0.07617	0.00449	0.97414
12	0.5588	0.4151	0.0261	0.5587	0.4153	0.0260	0.89097	0.10089	0.00814	0.89095	0.10094	0.00810	0.96474
13	0.4696	0.4917	0.0387	0.4697	0.4918	0.0385	0.85054	0.13576	0.01370	0.85059	0.13578	0.01363	0.95584
14	0.3724	0.5647	0.0629	0.3725	0.5648	0.0627	0.79102	0.18285	0.02612	0.79112	0.18283	0.02605	0.94528
15	0.2529	0.6244	0.1227	0.2526	0.6251	0.1224	0.67970	0.25582	0.06448	0.67934	0.25630	0.06437	0.93008
16	0.1956	0.6296	0.1748	0.1949	0.6309	0.1741	0.60045	0.29463	0.10491	0.59952	0.29578	0.10470	0.92160
17	0.1434	0.6245	0.2321	0.1429	0.6255	0.2316	0.50497	0.33524	0.15980	0.50394	0.33633	0.15973	0.91550
18	0.1113	0.6060	0.2827	0.1109	0.6065	0.2825	0.42981	0.35675	0.21345	0.42896	0.35746	0.21358	0.90986
19	0.0665	0.5324	0.4011	0.0660	0.5329	0.4010	0.29414	0.35899	0.34687	0.29257	0.36000	0.34743	0.89917
20	0.0503	0.4831	0.4666	0.0500	0.4832	0.4668	0.23377	0.34226	0.42397	0.23246	0.34282	0.42472	0.89400
21	0.0321	0.4036	0.5644	0.0319	0.4036	0.5645	0.15737	0.30163	0.54099	0.15670	0.30186	0.54144	0.88752
22	0.0141	0.2929	0.6930	0.0141	0.2927	0.6933	0.07259	0.22987	0.69754	0.07236	0.22971	0.69793	0.88013
23	0.00600	0.20271	0.79129	0.00598	0.20272	0.79130	0.03131	0.16127	0.80741	0.03121	0.16130	0.80749	0.87499
24	0.0012	0.1011	0.8976	0.0012	0.1012	0.8976	0.00625	0.08023	0.91353	0.00644	0.08025	0.91331	0.87032
25	0.00038	0	0.99962	0.00034	0	0.99966	0.00194	0.00000	0.99806	0.00194	0	0.99806	0.86691
26	n/a	n/a	n/a	0.0005^b^	0	0.9995^b^	n/a	n/a	n/a	0.00255	0	0.99745	n/a

a Type B standard uncertainties *u* for temperature and pressure are *u*(*T*)=0.03 K and *u*(*p*)=0.002 MPa. The maximum error in density measurement is 5‧10^−5^ g/mL. For three-component samples (points # 2–24), combined standard uncertainties for mass fractions of H_2_O, BEG, and TOL are 0.0008, 0.0008, and 0.0007, respectively. In case of the two-component sample (point 1) combined standard uncertainty for mass fractions of H_2_O and TOL is 0.0004. For the two-component sample (point 25) combined standard uncertainty for mass fractions of H_2_O and TOL are 0.0002.

b Literature data on solubility of water in toluene [10].

**Table 2:** . Phase separation boundary on H_2_O–BEG–TOL system at *T*=293.15 K, *p*=0.100 MPa and approximation residuals.

	One-phase points		
Point number	X	Y	Y residuals
I	II	III	IV
1	0.001	0	0.000823
2	0.0127	0.0207	-0.000372
3	0.0236	0.0402	-0.000548
4	0.0415	0.0711	-0.000329
5	0.0501	0.0855	-0.000290
6	0.0586	0.0994	-0.000291
7	0.0865	0.1452	0.000826
8	0.1186	0.1955	0.000963
9	0.1388	0.2254	-0.000419
10	0.1967	0.3141	0.000310
11	0.2185	0.3465	0.000549
12	0.2698	0.4153	-0.002843
13	0.3284	0.4918	0.000279
14	0.3985	0.5648	0.002594
15	0.5022	0.6251	0.000661
16	0.5653	0.6309	-0.004233
17	0.6286	0.6255	0.000472
18	0.6764	0.6065	0.002489
19	0.7708	0.5329	0.000522
20	0.818	0.4832	-0.000763
21	0.8848	0.4036	-0.001300
22	0.9695	0.2927	0.001037
23	1.0308	0.2027	0.000242
24	1.0949	0.1012	-0.000527
25	1.1541	0	0.000148

Y is mass fraction of hydrotrope *ω*_h_, $X=\left(2\;\times \;\omega_{\text{TOL}}\;+\;\omega_{h}\right)/\sqrt{3}$.

Y residuals is difference between experimental and fitted value (9th order polynomial) of mass fraction of hydrotrope.

**Table 3:** Composition and interfacial tension for H_2_O–BEG–TOL two-phase samples at 293.15 K.^a^

	Initial composition										
	Mass fraction			Mole fraction							
Sample	H_2_O	BEG	TOL	H_2_O	BEG	TOL	*x*_BEG_ in aqueous phase	*U*_c_ of *x*_BEG_ in aqueous phase	*ω*_BEG_ in aqueous phase	*γ*, 10^−3^‧N/m	*γ*/*γ*_0_
I	II	III	IV	V	VI	VII	VIII	IX	X	XI	XII
BEG0	0.5000	0	0.5000	0.8365	0.0000	0.1635	0	0	0.0000	34.5±0.3 (WP)^b^	1
BEG1	0.4927	0.0102	0.4971	0.8330	0.0026	0.1643	0.0015	0.00051	0.0097	18.70 ±0.02	5.421‧10^−1^
BEG2	0.4784	0.0302	0.4915	0.8261	0.0080	0.1660	0.0041	0.00061	0.0261	12.07±0.02	3.499‧10^−1^
BEG3	0.4417	0.0600	0.4983	0.8056	0.0167	0.1777	0.0073	0.00070	0.0461	8.97±0.01	2.601‧10^−1^
BEG4	0.4291	0.1200	0.4509	0.8012	0.0342	0.1646	0.0098	0.00078	0.0611	5.83±0.02	1.690‧10^−1^
BEG5	0.4251	0.1800	0.3949	0.8025	0.0518	0.1457	0.0116	0.00077	0.0714	4.225±0.006	1.225‧10^−1^
BEG6	0.4284	0.2600	0.3116	0.8099	0.0749	0.1152	0.0130	0.00076	0.0796	2.822±0.07	8.180‧10^−2^
BEG7	0.4502	0.3200	0.2298	0.8277	0.0897	0.0826	0.0149	0.00089	0.0901	1.895±0.004	5.494‧10^−2^
BEG8	0.4737	0.3600	0.1663	0.8442	0.0978	0.0579	0.0163	0.00063	0.0978	1.404±0.003	4.069‧10^−2^
BEG9	0.5018	0.3901	0.1081	0.8616	0.1021	0.0363	0.0188	0.00039	0.1116	0.765±0.001	2.218‧10^−2^
BEG10	0.5552	0.3701	0.0747	0.8866	0.0901	0.0233	0.0184	0.00039	0.1092	0.579±0.001	1.679‧10^−2^
BEG11	0.5987	0.3477	0.0536	0.9041	0.0800	0.0158	0.0193	0.00039	0.1142	0.444±0.002	1.288‧10^−2^
BEG12	0.6185	0.3300	0.0515	0.9111	0.0741	0.0148	0.0194	0.00035	0.1145	0.374±0.001	1.083‧10^−2^
BEG13	0.6291	0.3304	0.0405	0.9152	0.0733	0.0115	0.0218	0.00034	0.1271	0.294±0.001	8.514‧10^−3^
BEG14	0.6594	0.3133	0.0273	0.9255	0.0670	0.0075	0.0234	0.00032	0.1356	0.132±0.001	3.839‧10^−3^
BEG15	0.6746	0.3047	0.0207	0.9304	0.0641	0.0056	0.0253	0.00031	0.1453	0.1194±0.0001	3.461‧10^−3^
BEG16	0.6743	0.3055	0.0202	0.9303	0.0643	0.0054	0.0270	0.00038	0.1535	0.0481±0.0002	1.396‧10^−3^
BEG17	0.6772	0.3032	0.0196	0.9312	0.0636	0.0053	0.0275	0.00032	0.1562	0.0426±0.0001	1.236‧10^−3^
BEG18	0.6797	0.3018	0.0184	0.9320	0.0631	0.0049	0.0297	0.00036	0.1667	0.0236±0.0002	6.827‧10^−4^
BEG19	0.6820	0.3005	0.0175	0.9327	0.0626	0.0047	0.0318	0.00034	0.1767	0.0234±0.0001	6.784‧10^−4^
BEG20	0.6858	0.2984	0.0159	0.9338	0.0619	0.0042	0.0341	0.00037	0.1873	0.01241±0.00004	3.596‧10^−4^

a the standard deviation of interfacial tension is automatically calculated by Advance software (Krüss, Hamburg, Germany).

b WP is the Wilhelmy plate method.

**Table 4:** Density *ρ* and dynamic viscosity *η* for equilibrated phases of H_2_O–BEG–TOL two-phase samples at 293.15 K.^a^

	Aqueous phase							Oil phase							
	Composition in mole fraction			*U*_c_ of composition in mole fraction				Composition in mole fraction			*U*_c_ of composition in mole fraction				
Sample	H_2_O	BEG	TOL	BEG	TOL	*ρ*, g/mL	*η*, mPa∙s	H_2_O	BEG	TOL	BEG	TOL	*ρ*, g/mL	*η*, mPa∙s	*∆ρ*, g/mL
I	II	III	IV	V	VI	VII	VIII	IX	X	XI	XII	XIII	XIV	XV	XVI
	rest	0	0.00011^c^	n/a	0.000033^c^	n/a	1.002^b^	0.0024^c^	0	0.9976^c^	n/a	0.0008	n/a	0.588^b^	n/a
BEG0	rest	0	0.0002	n/a	0.0001	0.99819	1.002	0.0046	0	0.9954	n/a	0.0029	0.86691	0.588	0.13128
BEG1	rest	0.0015	0.0002	0.0005	0.0001	0.99796	1.035	rest	0.0136	0.9839	0.0019	0.0030	0.86746	0.604	0.13050
BEG2	rest	0.0041	0.0001	0.0006	0.0001	0.99757	1.092	rest	0.0300	0.9671	0.0095	0.0108	0.86780	0.613	0.12977
BEG3	rest	0.0073	0.0001	0.0007	0.0001	0.99718	1.162	rest	0.0573	0.9389	0.0016	0.0029	0.86927	0.666	0.12791
BEG4	rest	0.0098	0.0001	0.0008	0.0001	0.99690	1.226	rest	0.1384	0.8397	0.0015	0.0030	0.87352	0.772	0.12338
BEG5	rest	0.0116	0.0001	0.0008	0.0001	0.99671	1.271	rest	0.2107	0.7313	0.0018	0.0036	0.87857	0.898	0.11814
BEG6	rest	0.0130	0.0001	0.0008	0.0001	0.99655	1.321	rest	0.2995	0.5472	0.0028	0.0053	0.88723	1.286	0.10932
BEG7	rest	0.0149	0.0002	0.0009	0.0002	0.99636	1.367	rest	0.3534	0.3822	0.0024	0.0038	0.89678	1.799	0.09958
BEG8	rest	0.0163	0.0002	0.0007	0.0001	0.99618	1.415	rest	0.3640	0.2509	0.0019	0.0028	0.90640	2.442	0.08978
BEG9	rest	0.0188	0.0003	0.0004	0.0001	0.99559	1.545	rest	0.3119	0.1246	0.0020	0.0016	0.91902	3.329	0.07657
BEG10	rest	0.0184	0.0003	0.0004	0.0001	0.99571	1.486	rest	0.2746	0.0808	0.0015	0.0011	0.92603	3.791	0.06968
BEG11	rest	0.0193	0.0003	0.0004	0.0001	0.99544	1.510	rest	0.2435	0.0553	0.0013	0.0008	0.93276	4.055	0.06268
BEG12	rest	0.0194	0.0003	0.0004	0.0001	0.99542	1.511	rest	0.2504	0.0600	0.0013	0.0007	0.93132	4.062	0.06410
BEG13	rest	0.0218	0.0004	0.0004	0.0001	0.99457	1.782	rest	0.2186	0.0412	0.0013	0.0008	0.93779	4.244	0.05678
BEG14	rest	0.0234	0.0004	0.0003	0.0001	0.99390	1.732	rest	0.1776	0.0243	0.0011	0.0003	0.94641	4.053	0.04749
BEG15	rest	0.0253	0.0005	0.0003	0.0001	0.99310	1.799	rest	0.1521	0.0170	0.0011	0.0004	0.95214	4.320	0.04096
BEG16	rest	0.0270	0.0006	0.0004	0.0001	0.99240	1.845	rest	0.1436	0.0152	0.0011	0.0002	0.95406	4.304	0.03834
BEG17	rest	0.0275	0.0006	0.0003	0.0001	0.99218	1.793	rest	0.1403	0.0145	0.0010	0.0002	0.95481	4.285	0.03737
BEG18	rest	0.0297	0.0007	0.0004	0.0001	0.99126	1.902	rest	0.1310	0.0128	0.0010	0.0002	0.95692	4.225	0.03434
BEG19	rest	0.0318	0.0009	0.0003	0.0001	0.99038	2.012	rest	0.1221	0.0113	0.0013	0.0002	0.95896	4.175	0.03142
BEG20	rest	0.0341	0.0010	0.0004	0.0001	0.98940	1.910	rest	0.1090	0.0093	0.0009	0.0001	0.96227	4.117	0.02713
CP	0.9301	0.0663	0.0036	0.0003	0.0001	0.97744	-	0.9301	0.0663	0.0036	0.0003	0.0001	0.97744	-	0

a the maximum error in density measurement is 5‧10^−5^ g/mL, the maximum error is 0.5 % of the measured value for viscosity.

b viscosity of pure water [11] and toluene [12].

c literature data on solubility of toluene in water and water in toluene [10].

## 2 Experimental Design, Materials and Methods

For the preparation of samples of the ternary system toluene (99.9% purity determined by the manufacturer, Ecos-1, Russia), BEG (99.5% purity determined by the manufacturer, Ecos-1, Russia), and deionized water (≥18.1 MΩ‧cm, Simplicity UV, Millipore) were used. The samples were prepared in custom-made jacketed cells with an inverted ground joint using the freshly calibrated balances PA413C (resolution 0.001 g, maximum error ±0.01 g, Ohaus, USA).

The location of the binodal curve and the critical point on the diagram was determined by volumetric titration from a two-phase state (detected visually as turbidity of the sample) to a homogeneous solution at 293.15±0.10 K. The position of the critical point was determined by titration with a volumetric phase ratio of 1:1. The samples were titrated in two stages. First, two-component water-toluene samples were titrated with BEG. Then, new two-phase samples with compositions in the immediate proximity to the expected separation boundary were prepared and titrated using small amounts of the titrants. The solubilities of toluene in water and water in toluene were obtained by titrating two-component samples with water or toluene. The combined standard uncertainty (*U_c_*) was calculated according to JCGM 100:2008, taking into account the purity of the components, maximum errors of weighing, and dosing uncertainty of titrants [3]. The densities of homogeneous titrated samples were measured with density meter DMA 4500 (Anton Paar, Austria) to construct an interpolation of the density along the separation boundary. The maximum error in density measurement is 5‧10^−5^ g/mL.

The two-phase samples for density, viscosity, and interfacial tension measurements were magnetically stirred for at least 12 h at 293.15±0.1 K after preparation, and then they were equilibrated without stirring for at least 12 h for complete stratification of phases. The density and dynamic viscosity of equilibrated phases in the two-phase region were measured at 293.15±0.03 K using a rolling-ball viscometer Lovis 2000 ME (Anton Paar, Austria) integrated with a density meter DMA 4500. The maximum error is 0.5 % of the measured value for viscosity. The interfacial tension of two-phase samples was measured using a spinning drop tensiometer SDT (Krüss, Hamburg, Germany) equipped with a circulation thermostat, Ministat 230 (Huber, Offenburg, Germany). The interfacial tension between water and toluene was determined with a force tensiometer K20 (Krüss, Hamburg, Germany) equipped with a circulation thermostat, MPC-E (Huber, Offenburg, Germany), by the Wilhelmy plate method. The compositions of equilibrated phases were obtained through density-interpolation data collected during titration. The interfacial tension data were approximated by a crossover function [4], combining the Langmuir – von Szyszkowski isotherm and the near-critical behavior predicted by the scaling theory [5].

## Ethics Statements

The studies described in the manuscript were conducted adhering to Ethics in publishing standards (https://www.elsevier.com/journals/data-in-brief/2352-3409/guide-for-authors) and did not involve human or animal subjects.

## CRediT authorship contribution statement

**Alexandra A. Kuchierskaya:** Investigation, Data curation, Writing – original draft. **Anton P. Semenov:** Data curation, Visualization, Investigation, Writing – review & editing. **Adeliya R. Sayfutdinova:** Investigation. **Dmitry S. Kopitsyn:** Investigation. **Vladimir A. Vinokurov:** Supervision. **Mikhail A. Anisimov:** Supervision, Validation, Visualization, Writing – review & editing. **Andrei A. Novikov:** Supervision, Data curation, Validation, Writing – review & editing.

## Declaration of Competing Interest

The authors declare that they have no known competing financial interests or personal relationships that could have appeared to influence the work reported in this paper.
